# Unexpectedly high burden of rotavirus gastroenteritis in very young infants

**DOI:** 10.1186/1471-2431-10-40

**Published:** 2010-06-11

**Authors:** H Fred Clark, Amy E Marcello, Diane Lawley, Megan Reilly, Mark J DiNubile

**Affiliations:** 1 Division of Infectious Diseases, The Children's Hospital of Philadelphia (CHOP), University of Pennsylvania School of Medicine, Philadelphia, PA, USA; 2 Department of Medical Communications, Merck Research Laboratories, North Wales, PA, USA

## Abstract

**Background:**

The highest incidence of rotavirus gastroenteritis has generally been reported in children 6-24 months of age. Young infants are thought to be partially protected by maternal antibodies acquired transplacentally or via breast milk. The purpose of our study was to assess the age distribution of children with confirmed community-acquired rotavirus gastroenteritis presenting to an urban referral hospital.

**Methods:**

Children presenting to The Children's Hospital of Philadelphia with acute gastroenteritis have been monitored for the presence of rotavirus antigen in the stool by ELISA (followed by genotyping if ELISA-positive) since the 1994-95 epidemic season.

**Results:**

Over the last 12 rotavirus seasons prior to the introduction of the pentavalent rotavirus vaccine in 2006, stool specimens from 1646 patients tested positive for community-acquired rotavirus infection. Gender or age was not recorded in 6 and 5 cases, respectively. Overall, 58% of the cases occurred in boys. G1 was the predominant VP7 serotype, accounting for 72% of cases. The median (IQR) age was 11 (5-21) months. A total of 790 (48%) cases occurred in children outside the commonly quoted peak age range, with 27% in infants <6 months of age and 21% in children >24 months of age. A total of 220 (13%) cases occurred during the first 3 months of life, and the highest number of episodes per month of age [97 (6%)] was observed during the second month of life.

**Conclusions:**

The incidence of community-acquired rotavirus gastroenteritis monitored over 12 seasons in the prevaccine era at a major university hospital was nearly constant for each month of age during the first year of life, revealing an unexpectedly high incidence of symptomatic rotavirus disease in infants <3 months old. A sizeable fraction of cases occurred in children too young to have been vaccinated according to current recommendations.

## Background

In the absence of a safe and effective vaccine (1-3), rotavirus has consistently been the leading cause of dehydrating gastroenteritis in infants and young children around the world (4-20). Virtually all children are infected at least once within the first 5 years of life, with the peak incidence widely quoted as occurring between 6 and 24 months of age (5, 7, 11, 13, 14, 16, 20-23). For the first few months of life, infants are thought to be partially protected by maternal antibodies acquired transplacentally or through breast feeding (24-26). The Advisory Committee on Immunization Practices currently recommends the initiation of the rotavirus vaccine series at 2 months of age, although the first dose can be given as early as age 6 weeks (27, 28). If infants during the first few months of life are at greater risk for symptomatic rotavirus infection than has been generally appreciated, routine immunization schedules might be reconsidered in order to extend the benefits of rotavirus vaccine to these vulnerable infants. Of course, the safety, effectiveness, and feasibility of immunizing neonates would have to be established. We examined the age distribution of children presenting to The Children's Hospital of Philadelphia (CHOP) with community-acquired rotavirus gastroenteritis prior to the recent introduction of the new rotavirus vaccines to assess the burden of rotavirus infection serious enough to motivate hospital visits in neonates and young infants.

## Methods

The surveillance protocol was approved by the institutional review board at CHOP (1, 29, 30). Informed consent from legal guardians was not required to procure and process stool specimens already obtained from children with acute gastroenteritis as part of standard clinical practice. Based on prior experience, the rotavirus epidemic season in Philadelphia was defined as the seven-month period from December 1 through June 30 of the following year (1, 29, 31). For 14 consecutive rotavirus seasons (1994-95 to 2007-08), all patients (regardless of age) presenting to CHOP with acute gastroenteritis and having an adequate stool sample were tested by a commercial qualitative enzyme-linked immunosorbent assay (ELISA) for rotavirus antigen (Premier Rotaclone, Meridian Bioscience, Cincinnati, OH).

For the first 5 rotavirus seasons studied (1994-95 to 1998-99), a subset of ELISA-positive clinical samples were analyzed at the Centers for Disease Control (CDC) as part of a national surveillance program. Viruses of identical electropherotype to our samples serotyped at the CDC were considered the same as the reference type (32). Beginning with the 1999-2000 season, ELISA-positive specimens were submitted to Merck Research Laboratories (MRL) for genotyping by reverse transcriptase-polymerase chain reaction (RT-PCR) if the quantity of the stool sample permitted. For the MRL analysis, a 365-bp RT-PCR product targeting the VP7 gene was amplified from isolated RNA and subsequently sequenced (33). Designation of G-type was based on nucleic acid homology in comparison with a database of sequences of known serotypes.

For the present analysis, only community-acquired cases during the last 12 rotavirus seasons prior to the introduction of the pentavalent rotavirus vaccine (RV5; RotaTeq, Merck, Whitehouse Station, NJ, USA) in 2006 were analyzed. A monovalent rotavirus vaccine (RV1; Rotarix, GlaxoSmithKline Biologicals, Rixensart, Belgium) was approved in April 2008. A tetravalent vaccine (Rotashield, Wyeth, Collegeville, PA, USA) had been temporarily available in the United States in 1998-99 before being withdrawn from the market because of the risk of intussusception. Families seeking care at CHOP resided for the most part in Philadelphia or its immediately surrounding suburbs (34). Hospital-acquired infections, defined as developing >48 hours after admission or <48 hours after discharge, were excluded. Nosocomial infections had constituted ~20% of the total number of rotavirus cases diagnosed at CHOP in the pre-RV5 era (30). For each season and for all twelve seasons combined, the median (interquartile range [IQR]) age in months was calculated for children presenting to CHOP with community-acquired rotavirus gastroenteritis.

## Results

A total of 1646 unique community-acquired cases of rotavirus gastroenteritis were identified during 12 seasons from 1994-95 to 2005-06 (Table 1). Gender or age was not recorded in 6 and 5 cases, respectively. There were a higher percentage of boys than girls in each season, and overall there were 58% (n = 947) male cases and 42% (n = 693) female cases in the combined 12 monitored seasons (excluding the 6 cases where the gender was not recorded). The race for the majority of children when known was non-white. The predominant serotype was G1 (ranging from 41%-97%); however, the 1994-95 season had a high percentage of G3 cases (93%), the 1995-96 and 1999-2000 seasons had a high percentage of G9 cases (52% and 41%, respectively,) and the 2005-2006 season had a high percentage of G2 cases (39%).

**Table 1:** Demographic characteristics of the 1646 community-acquired cases of rotavirus gastroenteritis presenting to CHOP in the pre-vaccine era^◊ ^between the 1994-95 and 2005-06 epidemic seasons

Rotavirus season	Number of cases	**Median age**^**§ **^**[IQR] (months)**	**Predominant serotype**^**‡**^	**% male**^¶^	% urban residents	**% non-white race**^#^
1994-1995	178	8 [4 - 16]	G3	61%	majority unknown	47% 28% unknown
1995-1996	155	9 [4 - 16]	G9, G2	58%	70% 5% unknown	78% 2% unknown
1996-1997	115	9 [6 - 17.25]	G1	66%	majority unknown	52% 11% unknown
1997-1998	84	11.5 [6 - 24]	G1	55%	majority unknown	26% 49% unknown
1998-1999	166	10 [4 - 19.5]	G2, G1	57%	majority unknown	59% 11% unknown
1999-2000	94	11 [4.75 - 22]	G1 (47%) G9 (41%)	53%	66%	67%
2000-2001	115	14 [7 - 22]	G1 (95%)	62%	79%	50%
2001-2002	92	11 [6.25 - 19]	G1 (94%)	58%	44%	58%
2002-2003	29^†^	11 [5.5 -17]	G1 (97%)^†^	55%	48%	55%
2003-2004	158	13 [7 - 26.25]	G1 (69%) G12 (11%)	52%	47%	49%
2004-2005	185	14 [7 - 22]	G1 (87%)	53%	48%	55%
2005-2006	275	11 [5 - 25]	G1 (51%) G3 (39%)	59%	55%	70%

^**◊**^A tetravalent rotavirus vaccine had been transiently available in the United States in 1998-99.

^**§**^Age was not recorded for 5 children. The median [IQR] age for the other 1641 children seen at CHOP during the 12 rotavirus epidemic seasons combined was 11 (5 - 21) months.

^**‡**^From 1994-95 to 1998-99, serotypes were based on electropherotypes compared to CDC standards. Starting with the 1999-2000 season, serotypes were determined by RT-PCR genotyping.

^¶^Excludes 6 children where the gender was not recorded.

^#^The "non-white" category potentially includes children of African, Asian, Hispanic, Eskimo, and Native American heritage.

^†^Due to a laboratory accident, only 29 specimens from the 175 cases in the 2002-2003 rotavirus season were suitable for genotyping.

**Figure 1:**
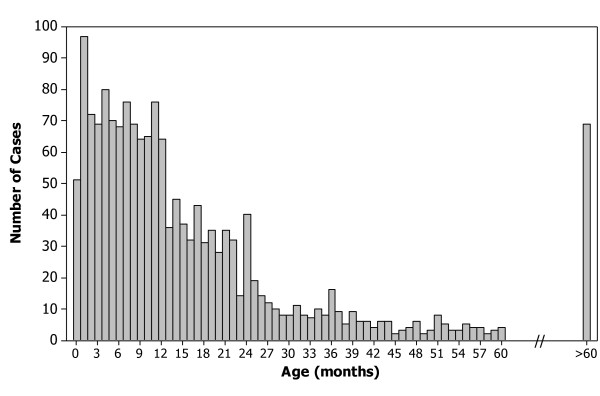
**Age distribution of the 1641 evaluable community-acquired rotavirus cases presenting to CHOP during the last 12 rotavirus epidemic seasons (1994-05 through 2005-06) prior to introduction of the new rotavirus vaccines**. A histogram shows the frequency of community-acquired rotavirus cases presenting to CHOP by age for the entire 12-year time period under study. Mean age = 18.1 months; standard deviation = 25.1 months; minimum age = 0 months; Q1 = 5 months; median age = 11 months; Q3 = 21 months; maximum age = 240 months; IQR = 16 months. Age was not recorded for 5 of the 1646 children with community-acquired rotavirus gastroenteritis presenting to CHOP during this period, who were therefore excluded from the analysis.

**Figure 2:**
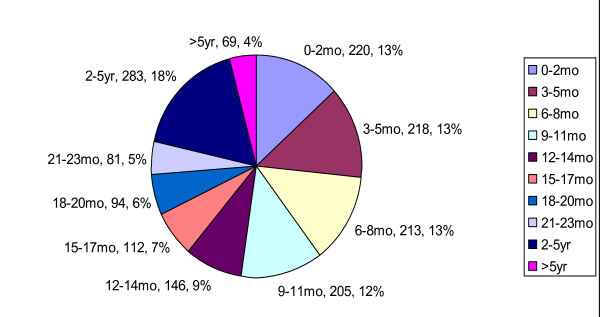
**A pie chart depicting the incidence of community-acquired rotavirus cases presenting to CHOP during the last 12 rotavirus epidemic seasons (1994-05 through 2005-06) prior to introduction of the new rotavirus vaccines**. Each slice is identified by age grouping, number of cases in the specified age range, and percentage of cases in the specified age range.

The median [IQR] age for the 1641 evaluable cases over the combined 12 rotavirus epidemic seasons prior to the availability of RV5 was 11 [5-21] months, with a full range of 0-240 months (Figure 1). Overall, 48% of children fell outside the commonly quoted peak age range of 6-24 months, with 27% of cases occurring in infants <6 months of age and 21% of cases occurring in children >24 months of age (Figure 2). A total of 220 (13%) cases occurred during the first 3 months of life, and the highest number of episodes per month of age [97 (6%)] were observed during the second month of life. In contrast to the prevaccine era, the median age of children presenting to CHOP with community-acquired rotavirus gastroenteritis during the 2007-08 season was 20 months and during the 2008-09 season was 23.5 months.

The serotype distribution by age group is displayed in Table 2. Despite some fluctuation in the predominant circulating serotype in the community, the age distribution was generally similar for each of the 12 seasons. However, for the two most common VP7 types, G1 cases occurred slightly less often in children <6 months of age (63%) than in older children (>70%), whereas G2 cases occurred twice as frequently in children during the first 6 months of life (19%) compared to children during the second 6 months of life (9%). Although the number of cases was relatively small, G9 rotaviruses were also disproportionately represented in children under the age of 6 months (11%) versus older children (<7%).

**Table 2:** Serotype of community-acquired rotavirus cases presenting to CHOP during the 6 rotavirus epidemic seasons (1999-2000 through 2005-06)^§ ^prior to introduction of the new rotavirus vaccines^◊ ^by age

	0 to 5 months	6 to 11 months	12 to 17 months	18 to 23 months	≥2 years	All Ages
	
	n (%)	n (%)	n (%)	n (%)	n (%)	n (%)
**G1**	134 (63)	182 (76)	112 (74)	90 (78)	154 (71)	672 (72)
**G2**	41 (19)	20 (9)	17 (11)	12 (10)	39 (18)	129 (14)
**G3**	3 (1)	6 (3)	5 (3)	0 (0)	3 (1)	17 (2)
**G4**	6 (3)	3 (1)	4 (3)	0 (0)	5 (2)	18 (2)
**G8**	0 (0)	1 (<1)	0 (0)	0 (0)	0 (0)	1 (<1)
**G9**	24 (11)	15 (6)	8 (5)	7 (6)	13 (6)	67 (7)
**G12**	5 (2)	11 (5)	6 (4)	6 (5)	2 (1)	30 (3)

**Totals **(7 seasons)	**213 (100)**	**238 (100)**	**152 (100)**	**115 (100)**	**216 (100)**	**934 (100)**

n (%), number (percent) of cases in the specified age category infected with each identified rotavirus serotype.

^§^Starting with the 1999-2000 season, ELISA-positive specimens were genotyped by reverse transcriptase-polymerase chain reaction at Merck Research Laboratories when the quantity of stool specimen permitted (1, 33). Designation of G-serotype was based on nucleic acid homology in comparison with a database of sequences of known serotypes.

^**◊**^A tetravalent rotavirus vaccine had been transiently marketed in the United States in 1998-99.

## Discussion

Although rotavirus accounts for an estimated half-million deaths each year in the developing world (17), the public health burden of rotavirus gastroenteritis in industrialized nations is largely measured in infant morbidity, healthcare costs, missed daycare, and loss of time from work for parents/guardians (6, 9, 11, 13, 15, 16, 19, 23, 35, 36). The rotavirus epidemic season in the United States typically begins in the autumn in the Southwest and spreads across the country ending in the spring of the following calendar year in the Northeast (31). Rotavirus infections typically peak in Philadelphia during March and April (1, 29, 35).

The general consensus dating back to the seminal report of a "reovirus-like agent" as the major cause of "winter" gastroenteritis in hospitalized infants and young children has been that symptomatic rotavirus infections predominantly afflict children between 6 and 24 months of age (14). Despite published data to the contrary (4, 9, 10, 15, 37, 38), respected textbooks of infectious diseases (5, 20) continue to propagate the dogma that rotavirus gastroenteritis is largely a disease of children older than 6 months of age through their second birthday. Assuming that the age distribution of the population served by CHOP is not skewed, we found that the monthly incidence of community-acquired rotavirus cases during the 12 years prior to licensing of RV5 was consistently high from birth to 12 months of age, followed by a marked and sustained decline in children older than 1 year. By compiling incidence data for individual months of age rather than for clusters, a high rotavirus attack rate was evident during the first 3 months of life. The highest number of episodes per month of age was seen among children in their second month of life. The surprising frequency of neonatal cases demonstrates that a non-trivial number of newborns might not be adequately protected by maternal antibodies (25, 26). The clear excess of rotavirus disease during the first versus second year of age in our experience also conflicts with several studies where more rotavirus disease was reported in the second year rather than the first year of life (9, 11, 12, 23).

Some differences between our data and earlier reports are more apparent than real. For example, the benchmark article by Kapikian *et al*. identified "reovirus" in stool specimens by electron microscopy from 60 of 143 tested children hospitalized with "winter" gastroenteritis, a not inconsequential 10 (17%) of whom were <6 months of age (14). Although the proportion of cases with "reovirus" identified in stool samples was threefold higher in children 6-12 months old [25 of the 39 children tested (64%)] than in younger infants [10 of the 48 children tested (21%)] hospitalized in the winter for acute gastroenteritis, these data are often misconstrued as establishing that symptomatic rotavirus infection before age 6 months is rare in an absolute sense. Other seeming inconsistencies between our findings and previously published data might be explained by selection and ascertainment biases, racial and socioeconomic factors, variations in the circulating serotypes, and/or geographical differences. As an illustration, for rotavirus gastroenteritis in the United States, hospitalization of black infants tends to occur on average at a younger age than hospitalization of white infants (7, 16). In addition, G2 rotavirus infections at CHOP occurred twice as frequently in children <6 months of age than in children 6-12 months of age. To further complicate direct comparisons of our results with prior studies indicating that infection with rotavirus in young infants requiring a healthcare intervention is not uncommon (4, 37, 38), some publications do not clearly distinguish nosocomial from community-acquired acquisition.

The methodological limitations of our analysis should be acknowledged. We did not directly assess the population incidence of severe rotavirus gastroenteritis. Instead, we used the number of children presenting to CHOP with acute gastroenteritis associated with a rotavirus-positive ELISA as our denominator. The index of suspicion for rotavirus infection might be influenced by the age of the child, and stool specimens may be more or less likely to be obtained from diapered babies than older children. The age distribution for community-acquired cases presenting to the hospital may be skewed toward younger age groups relative to the overall distribution in the community because infants may be more compromised by gastroenteritis than older children and/or parents may be more likely to seek medical attention for younger than for older children. Even though our approach may be confounded by selection bias, the sample is likely representative of the general population of children sick enough to necessitate a hospital visit for community-acquired rotavirus infection. While admittedly not definitive, these results indicate a need to re-examine the conventional wisdom and its implications for clinical practice as we move forward in the rotavirus vaccine era.

## Conclusions

The peak incidence of serious rotavirus infections is still generally quoted as occurring in children after 6 months of age and extending out to 24 months of age (5, 20). Although true in a cumulative sense, the monthly incidence of community-acquired cases seen at our hospital was comparable for both halves of the first year of life, and then dramatically dropped off. Based on 12 rotavirus seasons monitored at CHOP prior to the introduction of the recently licensed vaccines, we found a large fraction (48%) of cases outside the putative peak age range of 6-24 months. At CHOP from 1994-95 to 2005-06, the highest monthly incidence of rotavirus gastroenteritis was observed during the second month of life, by which time levels of maternal antibody against rotavirus may have substantially waned in many infants (25). Widespread uptake of an effective rotavirus vaccine could reduce maternal antibody levels by inducing herd immunity and reducing adult infections. On the other hand, herd immunity might serve to prevent infection in yet-to-be-vaccinated infants. Rotavirus vaccines are usually given to infants at ~2 months of age; even 1 dose of either the monovalent (RV1) or pentavalent (RV5) vaccine likely affords some protection against severe disease within 2 weeks of administration bridging the gap until the next dose. Subsequent to the introduction of the new rotavirus vaccines, the relative frequencies of cases in children both younger than 6 months and older than 2 years have increased at CHOP (1). Infants in the first few months of life may remain vulnerable to serious rotavirus infections and constitute a sizeable fraction of the cases seen at CHOP. Our findings should prompt a possible rethinking of the optimal rotavirus immunization schedule in order to safely and effectively balance the heretofore underappreciated susceptibility of newborns with concerns about suboptimal vaccine responses in the presence of maternal antibodies (28, 39-46).

## Competing interests

HFC is the co-inventor of the pentavalent rotavirus vaccine and serves as an investigator and consultant for Merck. As a current employee of Merck, MJD owns stock options in the company.

## Authors' contributions

The CHOP rotavirus surveillance program was established by HFC and jointly managed with DL. AEM and MR helped to collect data. MDN assisted with interpretation of the results and drafted the report with HFC and DL. All authors have made substantive contributions to the analysis, reviewed multiple iterations of the paper, and approved an essentially final version of the revised manuscript.

## Pre-publication history

The pre-publication history for this paper can be accessed here:

http://www.biomedcentral.com/1471-2431/10/40/prepub

## References

[B1] Clark HF Lawley DA Mallette LA DiNubile MJ Hodinka RL Decline in cases of rotavirus gastroenteritis presenting to The Children's Hospital of Philadelphia after introduction of a pentavalent rotavirus vaccine Clin Vaccine Immunol 2009 16 382 86 10.1128/CVI.00382-08 19158283 PMC2650872

[B2] Ruiz-Palacios GM Perez-Schael I Velazquez FR Safety and efficacy of an attenuated vaccine against severe rotavirus gastroenteritis N Engl J Med 2006 354 11 22 10.1056/NEJMoa052434 16394298

[B3] Vesikari T Matson DO Dennehy P Safety and efficacy of pentavalent human-bovine (WC3) reassortant rotavirus vaccine in preventing rotavirus gastroenteritis and reducing associated healthcare resource utilization N Engl J Med 2006 354 23 33 10.1056/NEJMoa052664 16394299

[B4] Cicirello HG Das BK Gupta A High prevalence of rotavirus infection among neonates born at hospitals in Delhi, India: predisposition of newborns for infection with unusual rotavirus Ped Infect Dis J 1994 13 720 724 10.1097/00006454-199408000-00008 7970972

[B5] Dormitzer PR Rotaviruses Mandel, GL, Bennett, JE, and Dolin R Principles and Practice of Infectious Diseases 2005 Sixth Elsevier, Churchill-Livingstone. Philadelphia 1902 1913 Chapter 146

[B6] Fischer TK Incidence of hospitalizations due to rotavirus gastroenteritis in Denmark Acta Paediatr 2001 90 1073 1075 10.1080/080352501316978183 11683198

[B7] Fischer TK Viboud C Parashar U Hospitalizations and deaths from diarrhea and rotavirus among children <5 years of age in the United States, 1993-2003 J Infect Dis 2007 195 1117 1125 10.1086/512863 17357047

[B8] Ford-Jones EL Wang E Petric M Corey P Moineddin R Fearon M Rotavirus-associated diarrhea in outpatient settings and child care centers. The Greater Toronto Area/Peel Region PRESI Study Group. Pediatric Rotavirus Epidemiology Study for Immunization Arch Pediatr Adolesc Med 2000 154 586 593 10850505 10.1001/archpedi.154.6.586

[B9] Forster J Guarino A Parez N Hospital-based surveillance to estimate the burden of rotavirus gastroenteritis among European children younger than 5 years of age Pediatrics 2009 123 e393 e400 10.1542/peds.2008-2088 19254975

[B10] Georges-Courbot MC Monges J Beraud-Cassel AM Gouandjika I Georges AJ Prospective longitudinal study of rotavirus infections in children from birth to two years of age in Central Africa Ann Inst Pasteur Virol 1988 139 421 428 10.1016/S0769-2617(88)80077-7 3214595

[B11] Gil A Carrasco P Jimenez R San Martin M Oyaguez I Gonzalez A Burden of hospitalizations attributable to rotavirus infection in children in Spain, period 1999-2000 Vaccine 2004 22 2221 2225 10.1016/j.vaccine.2003.11.037 15149780

[B12] Grimwood K Huang QS Cohet C Rotavirus hospitalisation in New Zealand children under 3 years of age J Paediatr Child Health 2006 42 196 203 10.1111/j.1440-1754.2006.00829.x 16630321

[B13] Jin S Kilgore PE Holman RC Clarke MJ Gangarosa EJ Glass RI Trends in hospitalizations for diarrhea in United States children from 1979 through 1992: estimates of the morbidity associated with rotavirus Pediatr Infect Dis J 1996 15 397 404 10.1097/00006454-199605000-00004 8724060

[B14] Kapikan AZ Kim HW Wyatt RG Human reovirus-like agent as the major pathogen associated with "winter" gastroenteritis in hospitalized infants and young children N Engl J Med 1976 294 965 972 176586 10.1056/NEJM197604292941801

[B15] Lynch M O'Halloran F Whyte D Fanning S Cryan B Glass RI Rotavirus in Ireland: national estimates of disease burden, 1997 to 1998 Pediatr Infect Dis J 2001 20 693 698 10.1097/00006454-200107000-00010 11465842

[B16] Malek MA Curns AT Holman RC Diarrhea- and rotavirus-associated hospitalizations among children less than 5 years of age: United States, 1997 and 2000 Pediatrics 2006 17 1887 1892 10.1542/peds.2005-2351 16740827

[B17] Parashar UD Hummelman EG Bresee JS Miller MA Glass RI Global illness and deaths caused by rotavirus disease in children Emerg Infect Dis 2003 9 565 572 12737740 10.3201/eid0905.020562 PMC2972763

[B18] Patel M Pedreira C De Oliveira LH Association between pentavalent rotavirus vaccine and severe rotavirus diarrhea among children in Nicaragua JAMA 2009 301 2243 2251 10.1001/jama.2009.756 19491186

[B19] Rodriguez WJ Kim HW Brandt CD Rotavirus gastroenteritis in the Washington, DC, area: incidence of cases resulting in admission to the hospital Am J Dis Child 1980 134 777 779 6250399 10.1001/archpedi.1980.02130200047015

[B20] Ward RL Bernstein DI Staat MA Rotaviruses Feigin RD, Cherry JD, Demmler-Harrison GJ, Kaplan SL Textbook of Pediatric Infectious Diseases 2009 Sixth 2245 2270 Chapter 185

[B21] Bettencourt JA Arbor E Malysz AS Oravec R Dias C Seasonal and age distribution of rotavirus infection in Porto Alegre-Brazil Braz J Infect Dis 2000 4 279 283 11136524

[B22] Rivest P Proulx M Lonergan G Lebel MH Bedard L Hospitalizations for gastroenteritis: the role of rotavirus Vaccine 2004 22 2013 2017 10.1016/j.vaccine.2003.10.029 15121314

[B23] Rodriguez WJ Kim HW Brandt CD Longitudinal study of rotavirus infection and gastroenteritis in families served by a pediatric medical practice: clinical and epidemiologic observations Pediatr Infect Dis J 1987 6 170 176 10.1097/00006454-198702000-00006 3031575

[B24] Duffy LC Byers TE Riepenhoff-Talty M La Scolea LJ Zielezny M Ogra PL The effects of infant feeding on rotavirus-induced gastroenteritis: a prospective study Am J Public Health 1986 76 259 263 10.2105/AJPH.76.3.259 3004238 PMC1646548

[B25] Elias MM Distribution and titres of rotavirus antibodies in different age groups J Hyg (Lond) 1977 79 365 372 200676 10.1017/s0022172400053201 PMC2129953

[B26] Misra S Sabui TK Basu S A prospective study of rotavirus diarrhea in children under 1 year of age Clin Pediatr (Phila) 2007 46 683 688 10.1177/0009922807300700 17554140

[B27] Centers for Disease Control Prevention of rotavirus gastroenteritis among infants and children MMWR 2006 55 1 13

[B28] Centers for Disease Control Rotavirus vaccination coverage and adherence to the advisory committee on immunization practices (ACIP)-recommended vaccination schedule - United States, February 2006-May 2007 MMWR 2008 57 398 401 18418345

[B29] Clark HF Lawley DA Schaffer A Assessment of the epidemic potential of a new strain of rotavirus associated with the novel G9 serotype which caused an outbreak in the United States for the first time in the 1995-1996 season J Clin Microbiol 2004 42 1434 1438 10.1128/JCM.42.4.1434-1438.2004 15070985 PMC387540

[B30] Smith MJ Clark HF Lawley D The clinical and molecular epidemiology of community- and healthcare-acquired rotavirus gastroenteritis Pediatr Infect Dis J 2008 27 54 58 10.1097/INF.0b013e31814b279d 18162939

[B31] Torok TJ Kilgore PE Clarke MJ Holman RC Bresee JS Glass RI Visualizing geographic and temporal trends in rotavirus activity in the United States, 1991 to 1996 Pediatr Infect Dis J 1997 16 941 946 10.1097/00006454-199710000-00007 9380468

[B32] Gouvea V Ho MS Glass R Serotypes and electropherotypes of human rotavirus in the USA: 1987-1989 J Infect Dis 1990 162 362 367 2165108 10.1093/infdis/162.2.362

[B33] DiStefano D Kraiochkine N Mallette L Novel rotavirus VP7 typing assay using a one-step reverse transcriptase PCR protocol and product sequencing and utility of the assay for epidemiological studies and strain characterization, including serotype subgroup analysis J Clin Microbiol 2005 43 5876 5880 10.1128/JCM.43.12.5876-5880.2005 16333070 PMC1317171

[B34] Coffin SE Zaoutis TE Rosenquist AB Incidence, complications, and risk factors for prolonged stay in children hospitalized with community-acquired influenza Pediatrics 2007 119 740 748 10.1542/peds.2006-2679 17403845

[B35] Coffin SE Marchant J Sawyer M Impact of acute rotavirus gastroenteritis on pediatric outpatient practices in the United States Pediatr Infect Dis J 2006 25 584 589 10.1097/01.inf.0000220251.27595.74 16804426

[B36] Parashar UD Holman RC Bresee JS Epidemiology of diarrheal disease among children enrolled in four West Coast health maintenance organizations. Vaccine Safety Datalink Team Pediatr Infect Dis J 1998 17 605 611 10.1097/00006454-199807000-00006 9686726

[B37] Salinas B Gonzalez G Gonzalez R Escalona Materan M Schael IP Epidemiologic and clinical characteristics of rotavirus disease during five years of surveillance in Venezuela Pediatr Infect Dis J 2004 23 S161 S167 10.1097/01.inf.0000142465.25992.c3 15502696

[B38] Truant AL Chonmaitree T Incidence of rotavirus infection in different age groups of pediatric patients with gastroenteritis J Clin Microbiol 1982 16 568 569 6290533 10.1128/jcm.16.3.568-569.1982 PMC272414

[B39] Daskalaki I Spain CV Long SS Watson B Implementation of rotavirus immunization in Philadelphia, Pennsylvania: high levels of vaccine ineligibility and off-label use Pediatrics 2008 122 e33 e38 10.1542/peds.2007-2464 18595974

[B40] Goveia MG DiNubile MJ Dallas MJ Heaton PM Kuter BJ Efficacy of pentavalent human-bovine (WC3) reassortant rotavirus vaccine based on breastfeeding frequency Pediatr Infect Dis J 2008 27 656 658 10.1097/INF.0b013e318168d29e 18520448

[B41] Goveia MG Rodriguez ZM Dallas MJ Safety and efficacy of the pentavalent human-bovine (WC3) reassortant rotavirus vaccine in healthy premature infants Pediatr Infect Dis J 2007 26 1099 1104 10.1097/INF.0b013e31814521cb 18043445

[B42] Nguyen TV Yuan L Pa MS Jeong KI Gonzalez AM Iosef C Low titer maternal antibodies can both enhance and suppress B cell responses to a combined live attenuated human rotavirus and VLP-ISCOM vaccine Vaccine 2006 24 2302 2316 10.1016/j.vaccine.2005.11.043 16361002

[B43] Patel MM Clark AD Glass RI Broadening the age restriction for initiating rotavirus vaccination in regions with high rotavirus mortality: benefits of mortality reduction versus risk of fatal intussusception Vaccine 2009 27 2916 2922 10.1016/j.vaccine.2009.03.016 19428901

[B44] Shim E Feng Z Martcheva M Castillo-Chavez C An age-structured epidemic model of rotavirus with vaccination J Math Biol 2006 53 719 746 10.1007/s00285-006-0023-0 16915388

[B45] Vesikari T Karvonen A Forrest BD Hoshino Y Chanock RM Kapikian AZ Neonatal administration of rhesus rotavirus tetravalent vaccine Pediatr Infect Dis J 2006 25 118 22 10.1097/01.inf.0000199288.98370.71 16462287

[B46] Vesikari T Ruuska R Delem A Andre FE Neonatal rotavirus vaccination with RIT 4237 bovine rotavirus vaccine: a preliminary report Pediatr Infect Dis J 1987 6 164 169 10.1097/00006454-198702000-00005 3031574

